# P06 Think of hypophosphatasia if atypical fracture with persistent low ALP levels

**DOI:** 10.1093/rap/rkad070.027

**Published:** 2023-09-27

**Authors:** Afzal Latheef, Katie Moss

**Affiliations:** Whipps Cross University Hospital, Barts Health NHS Trust, London, United Kingdom; St George’s University Hospitals NHS Foundation Trust, London, United Kingdom

## Abstract

**Introduction:**

Hypophosphatasia (HPP), a rare, underdiagnosed metabolic genetic bone disease caused by diminished or absent expression of the tissue non-specific alkaline phosphatase (TNSALP) enzyme. The mutation of alkaline phosphatase (ALPL) gene leads to wide-ranging clinical manifestations, broadly classified into six different types based on age of onset and severity of the disease. Fragility fractures are predominant characteristics of adult onset HPP and are often misdiagnosed as osteoporosis, stipulating antiresorptive treatment. However, bisphosphonates (BP) make HPP more susceptible to atypical fractures and thus are contraindicated. A recent study shows 1:4 atypical femoral fracture cases are linked with monogenic bone diseases including HPP.

**Case description:**

A 63-year-old Caucasian female was referred to our osteoporosis clinic, at the age of 57 following an atypical right femur fracture while on bisphosphonate and steroids. Her past medical history includes multiple right thigh sarcoma at the age of 39, 45 and 49 years requiring multiple surgeries and localised radiotherapy. She was also diagnosed with ocular myasthenia gravis two years before the atypical fracture, requiring long term high dose steroids. Her other co-morbidities include type 2 diabetes and hypertension. Due to long term steroids, her primary care physician initiated weekly 70 mg oral alendronic acid. However, three months later she had a spontaneous right mid shaft femur fracture. The patient experienced one year of right thigh pain prior to fracture which was not brought to medical attention. She underwent intramedullary nailing and successful rehabilitation. A thorough assessment in fracture liaison service spotted the history of primary tooth loss with roots intact in childhood, and chronic dental problems crowning in adult life requiring many caps and crowns. Her mother has a history of “childhood onset rickets”, bowing of legs and complete loss of all her permanent teeth at the age of 30 years.

A review of blood tests revealed normal calcium, vitamin D and parathyroid levels, multiple low alkaline phosphatase levels between 16 – 23 U/L(normal range 30-130 U/L) prior to bisphosphonates, vitamin B6 fasting blood test had shown raised result at 140 (normal range 40-100). Xray of the left femur was normal. The dual energy X-ray absorptiometry (DEXA) scan yielded normal T score. The genetic test confirmed heterozygous gene mutation in ALPL gene, confirming a diagnosis of hypophosphatasia. She recovered well from her fracture and manages to walk more than one mile unaided. She is currently under regular follow-up in our hypophosphatasia clinic and has stopped bisphosphonates indefinitely.

**Discussion:**

The underlying cause for atypical femoral fracture (AFF) in this case is multifactorial; long-term steroids, radiotherapy, HPP and bisphosphonate usage. Regardless, there is a relationship between hypophosphatasia and AFF. Some studies have proved that more than 90% of the patients with AFF used long term BPs. The defective ALPL gene in HPP affects bone mineralisation, resulting in accumulation of inorganic pyrophosphate (PPi), which impairs hydroxyapatite crystal formation causing recurrent fractures, osteopenia, delayed wound healing, dental problems, atypical and pseudo fractures. Bisphosphonates are chemical derivates of PPi, and thus inversely affect bone mineralisation especially in cases of TNSALP mutations and thus are contraindicated in HPP. The atypical fractures often may remain undiagnosed for a long time despite often prolonged prodromal pain occurring before the actual fracture. Careful clinical evaluation and plain X-ray will help to make the diagnosis. Hypophosphatasia related fractures mostly occur laterally in the subtrochanteric diaphysis. AFF cases often require surgical repair with intramedullary nailing. If an imminent atypical fracture is diagnosed early, prophylactic surgery is sometimes recommended. After three months of alendronic acid, bone turnover markers are usually suppressed, therefore despite the short time of alendronate exposure, this is likely to have contributed to the atypical femur fracture. Also, genetic test revealed inherited autosomal dominant trait of HPP in this case, enabled family screening and prevention of potential use of future bisphosphonates in affected family members.

**Key learning points:**

HPP presenting in adulthood may be more common than previously though with a recent study estimating prevalence of the mild HPP genotype to be 1:508, although not all cases will be symptomatic. A persistent low alkaline phosphatase together with a fragility or atypical fracture raises suspicion of hypophosphatasia. A thorough clinical evaluation, fasting vitamin B6 levels, urine phosphoethanol amine levels and ALPL genetic test will aid the diagnosis. Severe cases of hypophosphatasia are treated with asfotase alfa, enzyme replacement therapy, to prevent debilitating complications, while most mild and moderate cases are offered supportive management. Bisphosphonate treatment is contraindicated in hypophosphatasia. It is worth reviewing ALP levels prior to antiresorptive therapy in suspected cases, as bisphosphonates often reduce ALP levels, masking the diagnosis. High risk Osteoporosis cases should be discussed in a multidisciplinary setting with careful risk-benefit evaluation. Cases like this highlight the importance of early HPP diagnosis to avoid contraindicating treatments and their consequences.

